# Socioeconomic Diversity of the Matriculating US Medical Student Body by Race, Ethnicity, and Sex, 2017**-**2019

**DOI:** 10.1001/jamanetworkopen.2022.2621

**Published:** 2022-03-15

**Authors:** Arman A. Shahriar, Vikram V. Puram, Jonathan M. Miller, Varun Sagi, Lorenzo Adolfo Castañón-Gonzalez, Shailendra Prasad, Renée Crichlow

**Affiliations:** 1 University of Minnesota Medical School, Minneapolis; 2 Hennepin Healthcare Research Institute, Minneapolis, Minnesota; 3 Department of Family Medicine and Community Health, University of Minnesota, Minneapolis; 4 Codman Square Health Center, Boston, Massachusetts; 5 Department of Family Medicine, Boston University, Boston, Massachusetts

## Abstract

This survey study compares the socioeconomic composition of the 2017 to 2019 matriculating medical student body with that of the US population, by self-identified race and ethnicity and by sex.

## Introduction

Workforce diversity contributes to the quality of health care,^1^ yet for decades, medical students, like other postsecondary education students,^2^ have disproportionately hailed from high-income households. Significant attention has been rightfully directed toward racial-, ethnic-, and gender-based diversity in medical schools,^3^ with little mention of socioeconomic diversity, which is a less visible form of diversity. This survey study compares the socioeconomic composition of the 2017 to 2019 matriculating medical student body with that of the US population, by self-identified race and ethnicity and by sex.

## Methods

This survey study was deemed not human participants research by the University of Minnesota and therefore exempt from institutional review board review and informed consent. This study is reported following the American Association for Public Opinion Research (AAPOR) reporting guideline.

We included matriculating allopathic medical students who reported parental income on the Association of American Medical Colleges Matriculating Student Questionnaire (AAMC-MSQ) between 2017 and 2019 (overall response rates, 65%-71%). The comparison group included households who responded to the Census Bureau’s Current Population Survey Annual Social and Economic Supplement (CPS-ASEC) between 2016 and 2018.

Respondents were categorized into household income groups using national quintile-based income limits. For each race and ethnicity, medical student household income was compared with that of the general population. We report a representation index (RI) for each subgroup, defined as the ratio of proportions of that subgroup in the medical student body and in the general US population. Values above and below 1.0 indicate overrepresentation and underrepresentation in the medical student body, respectively. For χ^2^ tests, *P* values were 1-sided, and statistical significance was set at *P* < .05. Detailed methods are provided in the eAppendix in the Supplement.

## Results

Of 44 903 AAMC-MSQ respondents, 30 373 (67.6%) reported parental income, of which 50.5% belonged to the top quintile of households, 24.0% belonged to the top 5%, and 52.4% were women. Four racial and ethnic groups accounted for 93% of the sample: 21.3% non-Hispanic Asian students, 6.4% non-Hispanic Black students, 10.9% Hispanic students of any race, and 54.0% non-Hispanic White students. Included and excluded respondents were similar apart from survey year, because beginning in 2018, respondents could enter unknown for parental income. Overall, this study captured 46.4% of matriculating allopathic medical students.

The Figure compares the income composition of each racial and ethnic group between the medical student body and US population. The top 5% of households were consistently overrepresented (RI > 1.0) in medical schools compared with the general population (all: RI, 4.8 [24.0% vs 5.0%]; *P* < .001; Asian: RI, 2.3 [21.7% vs 9.5%]; *P* < .001; Black: RI, 5.3 [9.1% vs 1.7%]; *P* < .001; Hispanic: RI, 6.6 [14.7% vs 2.2%]; *P* < .001; White: RI, 4.8 [28.6% vs 5.9%]; *P* < .001). The top quintile was overrepresented (RI > 1.0) for each race and ethnicity, and the bottom 3 quintiles were consistently underrepresented (RI < 1.0) (Figure). Variability by sex was less pronounced (Table).

**Figure.**
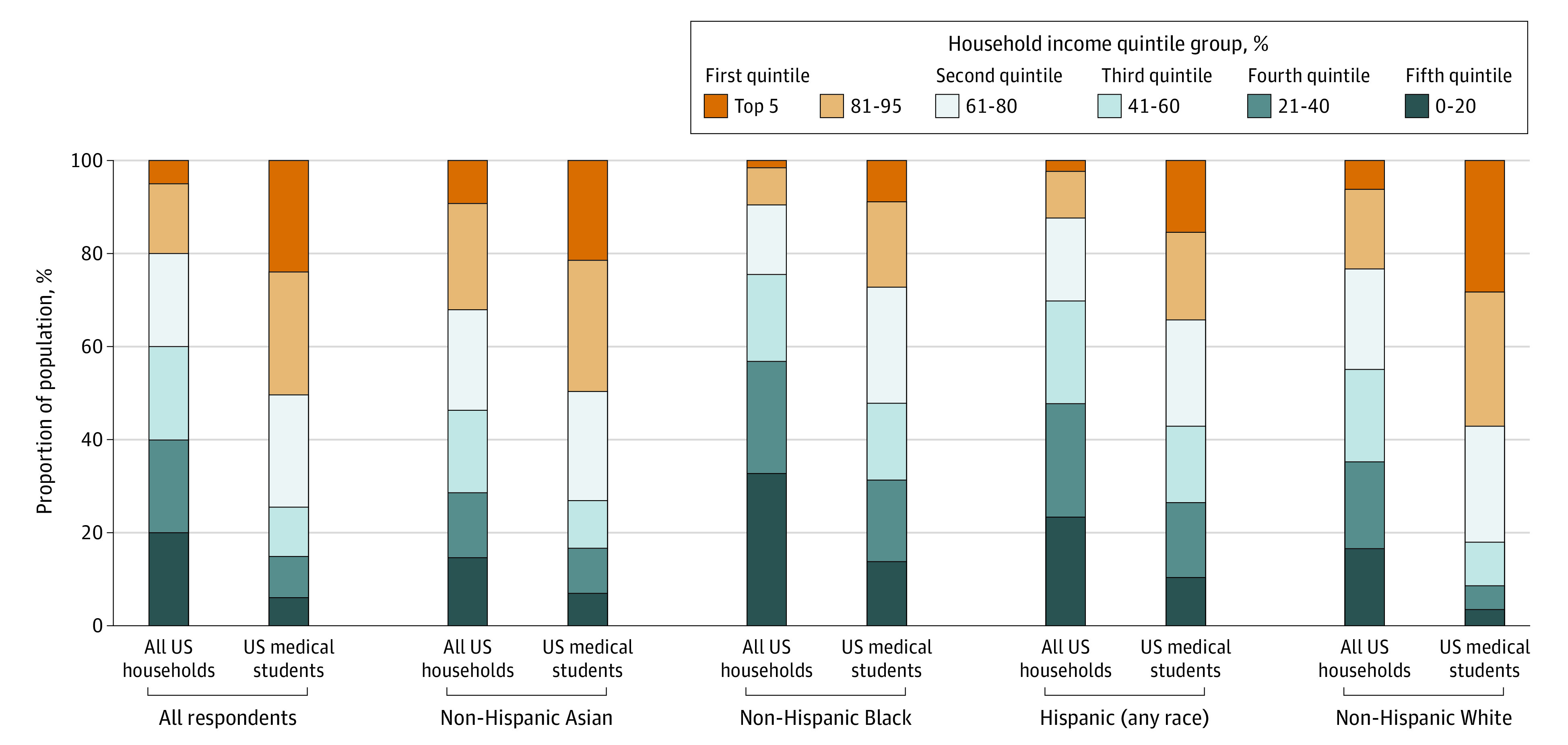
Comparison of Socioeconomic Composition of US Medical Students and US Households by 4 Major Racial and Ethnic Groups

**Table.** Household Income Distribution of Medical Students Compared With All US Households, by Race, Ethnicity, and Sex

Characteristic	Total, No.	Income percentile group, No. (%)^a^					
		Top 5%	81%-95%	61%-80%	41%-60%	21%-40%	Bottom 20%
**Race, ethnicity, and sex** ^b^							
All respondents							
All medical students	30 373	7302 (24.0)	8061 (26.5)	7325 (24.1)	3263 (10.7)	2578 (8.5)	1844 (6.1)
Sex							
Men	14 447	3403 (23.6)	3817 (26.4)	3460 (23.9)	1577 (10.9)	1269 (8.8)	921 (6.4)
Women	15 916	3898 (24.5)	4241 (26.6)	3861 (24.3)	1686 (10.6)	1308 (8.2)	922 (5.8)
All US households	382 475	19 128 (5.0)	57 367 (15.0)	76 495 (20.0)	76 495 (20.0)	76 495 (20.0)	76 495 (20.0)
RI	NA	4.81	1.77	1.21	0.54	0.42	0.30
Non-Hispanic Asian							
Medical students	6455	1403 (21.7)	1822 (28.2)	1496 (23.2)	640 (9.9)	640 (9.9)	454 (7.0)
Sex							
Men	3034	645 (21.3)	823 (27.1)	716 (23.6)	302 (10.0)	312 (10.3)	236 (7.8)
Women	3420	758 (22.2)	998 (29.2)	780 (22.8)	338 (9.9)	328 (9.6)	218 (6.4)
US households	20 123	1902 (9.5)	4542 (22.6)	4351 (21.6)	3515 (17.5)	2786 (13.8)	3026 (15.0)
RI	NA	2.30	1.25	1.07	0.57	0.72	0.47
Non-Hispanic Black							
Medical students	1959	179 (9.1)	355 (18.1)	483 (24.7)	323 (16.5)	342 (17.5)	277 (14.1)
Sex							
Men	721	71 (9.8)	120 (16.6)	156 (21.6)	120 (16.6)	143 (19.8)	111 (15.4)
Women	1237	108 (8.7)	235 (19.0)	327 (26.4)	203 (16.4)	199 (16.1)	165 (13.3)
US households	50 919	879 (1.7)	4082 (8.0)	7289 (14.3)	9660 (19.0)	12 260 (24.1)	16 749 (32.9)
RI	NA	5.29	2.26	1.72	0.87	0.73	0.43
Hispanic (any race)							
Medical students	3321	489 (14.7)	629 (18.9)	761 (22.9)	546 (16.4)	539 (16.2)	357 (10.7)
Sex							
Men	1648	224 (13.6)	315 (19.1)	348 (21.1)	294 (17.8)	272 (16.5)	195 (11.8)
Women	1673	265 (15.8)	314 (18.8)	413 (24.7)	252 (15.1)	267 (16.0)	162 (9.7)
US households	52 009	1167 (2.2)	5079 (9.8)	9309 (17.9)	11 440 (22.0)	12 733 (24.5)	12 281 (23.6)
RI	NA	6.56	1.94	1.28	0.75	0.66	0.46
Non-Hispanic White							
Medical students	16 407	4689 (28.6)	4712 (28.7)	4054 (24.7)	1522 (9.3)	848 (5.2)	582 (3.5)
Sex							
Men	7992	2204 (27.6)	2303 (28.8)	1999 (25.0)	750 (9.4)	448 (5.6)	288 (3.6)
Women	8409	2484 (29.5)	2409 (28.6)	2051 (24.4)	772 (9.2)	399 (4.7)	294 (3.5)
US households	253 820	14 951 (5.9)	42 976 (16.9)	54 649 (21.5)	50 747 (20.0)	47 541 (18.7)	42 957 (16.9)
RI	NA	4.85	1.70	1.15	0.46	0.28	0.21
**Disaggregation of non-Hispanic Asian medical students only**							
East Asian							
Chinese	1466	312 (21.3)	483 (32.9)	319 (21.8)	116 (7.9)	131 (8.9)	105 (7.2)
Korean	600	76 (12.7)	100 (16.7)	138 (23)	103 (17.2)	123 (20.5)	60 (10)
Taiwanese	294	59 (20.1)	74 (25.2)	88 (29.9)	37 (12.6)	16 (5.4)	20 (6.8)
Other^c^	183	43 (23.5)	55 (30.1)	53 (29.0)	14 (7.7)	12 (6.6)	6 (3.3)
Southeast Asian							
Filipino	209	36 (17.2)	67 (32.1)	70 (33.5)	21 (10.0)	9 (4.3)	6 (2.9)
Vietnamese	458	42 (9.2)	81 (17.7)	102 (22.3)	70 (15.3)	89 (19.4)	74 (16.2)
Other East Asian^d^	42	5 (11.9)	10 (23.8)	6 (14.3)	6 (14.3)	9 (21.4)	6 (14.3)
South Asian							
Pakistani	325	82 (25.2)	79 (24.3)	54 (16.6)	36 (11.1)	50 (15.4)	24 (7.4)
Bangladeshi	124	15 (12.1)	25 (20.2)	29 (23.4)	16 (12.9)	20 (16.1)	19 (15.3)
Indian	2129	638 (30.0)	693 (32.6)	496 (23.3)	141 (6.6)	94 (4.4)	67 (3.1)
Other South Asian^e^	33	8 (24.2)	9 (27.3)	8 (24.2)	3 (9.1)	3 (9.1)	2 (6.1)
Other Asian^e^	592	87 (14.7)	146 (24.7)	133 (22.5)	77 (13.0)	84 (14.2)	65 (11.0)

Abbreviations: NA, not applicable; RI, representation index.

^a^ Census household income limits (current dollars): 20%: $24 002 (2017), $24 882 (2018), $25 600 (2019); 40%: $45 600 (2017), $47 218 (2018), $50 000 (2019); 60%: $74 869 (2017), $77 150 (2018), $79 542 (2019); 80%: $121 018 (2017), $126 603 (2018), $130 000 (2019); 95%: $225 251 (2017), $244 088 (2018), $248 728 (2019). Limits from 2016, 2017, and 2018, as students were asked to estimate combined gross parental income for the previous year (eg, Association of American Medical Colleges Matriculating Student Questionnaire 2019 responses represent 2018 parental income). Proportions of medical students by income categories differed significantly from proportions of the general population by income categories for each race and ethnicity (standardized differences, >0.20; *P* < .001).

^b^ Race and ethnicity were categorized on the Association of American Medical Colleges Matriculating Student Questionnaire as White, non-Hispanic (includes students who self-identified as White); Black, non-Hispanic (includes students who self-identified as Black or African American, African, African American, Afro-Caribbean, and other Black or African American); Hispanic (any race) (includes students of any race who self-identified as Hispanic, Latino, or Spanish origin, Argentinean, Colombian, Cuban, Dominican, Mexican, Mexican American, Chicano/Chicana, Peruvian, Puerto Rican, and other Hispanic); Asian, non-Hispanic subgroups are mutually exclusive. Race and ethnicity for US households were based on response to the Current Population Survey Annual Social and Economic Supplement. Persons of Hispanic origin are determined based on a question asking if the person is Spanish, Hispanic, or Latino. If the response is yes, a follow-up question determines specific ethnic origin including the following options: Mexican, Mexican American, Chicano, Puerto Rican, Cuban, Cuban American, or some other Spanish, Hispanic, or Latino group. Race was self-selected from 6 defined race groups: White, Black or African American, American Indian or Alaskan Native, Asian, Native Hawaiian or Other Pacific Islander, and other.

^c^ Includes students who identified as East Asian, or multiple East Asian identities, such as both Japanese and Korean.

^d^ Includes students who identified as Indonesian, Cambodian, and Laotian.

^e^ The other South Asian and other Asian categories were self-selected by students that did not choose another category.

The 6 largest subgroups of Asian-identifying students were Indian (33.0%), Chinese (22.7%), Korean (9.3%), other Asian (9.2%), Vietnamese (7.1%), and Pakistani (5.0%). More than half (54.2%) of Chinese-identifying students were from the highest household income quintile. Likewise, 62.6% of Indian students were from the highest income quintile. More even income distributions were observed for medical students identifying as Korean, Vietnamese, Bangladeshi, and other Asian ethnicities.

## Discussion

In this exploratory survey study, high-income households were overrepresented in the medical student body both overall and within each racial and ethnic group. The underrepresentation of low-income groups was nearly ubiquitous across race and ethnicity groups.

Achieving demographic representation among physicians is a widely accepted ideal, but recent studies have shed light on the absence of progress with respect to race and ethnicity.^3^ Our findings suggest that underlying the lack of progress may be the inaccessibility of the profession to low-income students, who, owing to powerful historical and contemporary forces, like structural racism, are disproportionately students who identify as Black or Hispanic. A low socioeconomic status significantly decreases the likelihood that a student who is interested in medicine will apply or gain acceptance into medical school.^4^

Medical schools can assess socioeconomic disadvantage during the admissions process using essays and validated tools on the application server, like the parental education and occupation indicator. Likewise, common metrics, such as grade point average and Medical College Admission Test scores, can be adjusted for socioeconomic disadvantage.^1,5^ Long-term solutions will require upstream engagement, including community partnerships and targeted investments in pipeline programs.^1,4^ Matriculants who come from low-income households should be monitored for financial health and the accumulation of unexpected expenses, given that they do not have the family support of their peers from high-income households.^6^

This study has limitations. Reliability and validity of self-reported parental income are unknown, subject to response biases, and may differ between groups. Furthermore, AAMC-MSQ nonrespondent characteristics were unavailable, potentially introducing sampling variance. Future work should explore temporal trends and examine factors resulting in such a broad range of representation among the lowest-income students of various racial and ethnic groups.

## Notes

Supplement. **eAppendix.** Supplementary Methods
eReferences.
