# Supplementary material for: Socioeconomic Diversity of the Matriculating US Medical Student Body by Race, Ethnicity, and Sex, 2017-2019
Source: JAMA Netw Open. 2022 Mar 15;5(3):e222621. doi: 10.1001/jamanetworkopen.2022.2621 (PMC8924719; doi:10.1001/jamanetworkopen.2022.2621)
Supplement: Supplement. — eAppendix. Supplementary Methods eReferences. [file jamanetwopen-e222621-s001.pdf]

# Supplemental Online Content

Shahriar AA, Puram VV, Miller JM, et al. Socioeconomic diversity of the matriculating US medical student body by race, ethnicity, and sex, 2017-2019. *JAMA Netw Open*. 2022;5(3):e222621. doi:10.1001/jamanetworkopen.2022.2621

**eAppendix.** Supplementary Methods
**eReferences.**

This supplemental material has been provided by the authors to give readers additional information about their work.

© 2022 Shahriar AA et al. *JAMA Network Open.*

**eAppendix.** Supplementary Methods

Study Population

Medical students self-report their last years combined gross parental income on the AAMC-MSQ. Parental income reported on the AAMC-MSQ correlates strongly with parental education and occupation.[1,2] As medical students responding in 2017 to 2019 were reporting parent income for 2016 to 2018, the comparison group chosen was CPS-ASEC respondents from 2016 to 2018.[3]

We were unable to assess AAMC-MSQ non-respondent characteristics as these data were unavailable. Comparison between those included and excluded in our study was done using standardized differences, considering a threshold of < 0.20 (20%) as similar groups. We expected to exclude a larger percentage of 2018 and 2019 respondents, because beginning in 2018, respondents were given an option to enter "unknown" for parental income, resulting in fewer responses.[2]

Variables

*Income* - On the AAMC-MSQ, medical students reported combined gross parental income for the previous year, whereas CPS-ASEC respondents reported current household income.

*Race/Ethnicity* - Medical students and CPS respondents self-reported race and ethnicity from investigator-defined categories. For this study, respondents were categorized as Hispanic of any race, White non-Hispanic, Black non-Hispanic and Asian non-Hispanic. Race/ethnicity categories from the AAMC were heterogeneous (i.e., Asian, non-Hispanic), and details are included in the footnotes of **Figure 1.**

*Sex* - Medical students and CPS respondents self-reported sex as male or female.

Analysis

© 2022 Shahriar AA et al. *JAMA Network Open.*

The representation index (RI) for a given subgroup of students speaks to the degree of over- or underrepresentation of that subgroup. For example, "Top 5%" Black households comprised 9.1% of the Black medical student body, but 1.7% of Black US households. The RI is calculated as *0.091/0.017 = 5.3*. This is interpreted as the Top 5% being 5.3-times (430%) overrepresented in the medical student body.

For each race/ethnicity, AAMC-MSQ respondents were categorized into income groups based on the CPS-ASEC limits for the year corresponding to the MSQ response.[4] The CPS-ASEC reports the percent distribution of households by race/ethnicity within each income quintile and the top 5%; these publicly available data were downloaded and used for comparison,[5] and the exact thresholds are included in the footnotes of **Table 1.** Statistical comparisons between medical students and CPS respondents were done using chi-square tests and standardized differences. All analyses were conducted in 2021 with R version 3.6.1.

**AAPOR Standard Disclosure Checklist For Survey Research:**

| Basic Disclosure Element | Details |
|---|---|
| Survey sponsor | Association of American Medical Colleges |
| Survey/Data collection supplier | Association of American Medical Colleges |
| Population represented | 65-71% of matriculating U.S. medical students |
| Sample size | MSQ sample size 2017-2019: 44,903<br>Study sample size: 30,373 |
| Mode of data collection | Electronic survey; email |
| Type of sample | Census of all U.S. LCME-accredited medical schools |
| Start and end dates of data collection | MSQ 2017: 6/1/2017 - 9/15/2017<br>MSQ 2018: 6/1/2018 - 9/15/2018<br>MSQ 2019: 6/1/2019 - 9/15/2019 |
| Margin of sampling error for total sample | NA |
| Margin of sampling error for key subgroups | NA |
| Are the data weighted? | NA |
| Contact for more information | **Tyler Litsch, MPH, CHES**<br>Research and Data Analyst, AAMC<br>tlitsch@aamc.org |

© 2022 Shahriar AA et al. *JAMA Network Open.*

Questions Included for Analysis from the AAMC-MSQ 2017-2019

The following questions from the AAMC Matriculating Student Questionnaire (AAMC-MSQ) were included in this study. The questions are listed and numbered in the order in which they appear in the MSQ. All other questions are omitted from this Appendix.

** Indicates that the item is populated from other AAMC data sources (e.g., American Medical College Application Service [AMCAS])*

**1. Sex***

- Male
- Female

**2. How do you self-identify?*** ***Multiple responses allowed***

- American Indian or Alaska Native
- Asian
- Black or African American
- Hispanic, Latino, or Spanish origin
- Native Hawaiian or Other Pacific Islander
- White
- Other
- Non-U.S. citizen and non-permanent resident

**3. Regardless of your dependency status, please indicate your parents' combined gross income for last year (a rough estimate is sufficient). Note: starting in 2018, an additional response option of unknown parental income was added. Therefore, fewer participants reported parental income starting in 2018.**

- Free text

© 2022 Shahriar AA et al. *JAMA Network Open.*

**eReferences**

1. Youngclaus J, Roskovensky L. *Analysis in Brief: An Updated Look at the Economic Diversity of US Medical Students*. Association of American Medical Colleges; 2018.
2. Association of American Medical Colleges. Matriculating student questionnaire. 2017-2019. Accessed July 10, 2021. https://www.aamc.org/data-reports/students-residents/report/matriculating-student-questionnaire-msq
3. US Census Bureau Current Population Survey - Methodology. https://www.census.gov/programs-surveys/cps/technical-documentation/methodology.html. Published 2021. Accessed June 29, 2021.
4. US Census Bureau Current Population Survey. Income Distribution to $250,000 or More for Households. https://www.census.gov/data/tables/time-series/demo/income-poverty/cps-hinc/hinc-06.html. Published 2021. Accessed June 29, 2021.

US Census Bureau Current Population Survey. Percent Distribution of Households, by Selected Characteristics Within Income Quintile and Top 5 Percent. https://www.census.gov/data/tables/time-series/demo/income-poverty/cps-hinc/hinc-05.html. Published 2021. Accessed July 10, 2021.

© 2022 Shahriar AA et al. *JAMA Network Open.*
